# Screening Optimization of Latent Tuberculosis Infection in Rheumatoid Arthritis Patients

**DOI:** 10.1155/2015/569620

**Published:** 2015-07-29

**Authors:** Bella Mehta, Ekaterini Zapantis, Olga Petryna, Petros Efthimiou

**Affiliations:** ^1^Hospital for Special Surgery, New York, NY 10021, USA; ^2^North Shore-Long Island Jewish, Manhasset, NY 11030, USA; ^3^Beth Israel Medical Center, New York, NY 10003, USA; ^4^Rheumatology Division, New York Methodist Hospital, Weill Cornell Medical College, New York, NY 11215, USA

## Abstract

*Objective*. Rheumatoid arthritis (RA) patients are at increased risk of latent tuberculosis infection (LTBI) but there are no clear guidelines for LTBI screening with Tuberculin Skin Test (TST) or Quantiferon TB Gold testing (QFT-G). *Methods*. A retrospective study was conducted in a high risk, largely foreign-born, inner city, RA population. After screening 280 RA patients, 134 patients who had both TST and QFT-G testing performed during their initial evaluation were included. *Results*. Out of 132 RA patients included in our analysis, 50 (37.8%) patients were diagnosed with LTBI with either positive TST 42 (31.8%) or QFT-G 23 (17.4%). 15 (11.4%) were positive and 82 (62.1%) were negative for both tests. The agreement between TST and QFT-G was 73.5% (Kappa 0.305, CI = 95% 0.147–0.463, *p* = 0.081).  *Conclusions*. There was low-moderate agreement (*κ* = 0.305) between TST and QFT-G. In the absence of clearly defined gold standard and limitations associated with both tests, we propose early screening with both tests for patients who need prompt treatment with BRMs. Patients who are not immediate candidates for BRM treatment may be safely and cost effectively screened with a two-step process: initial screening with TST and if negative, IGRA testing. Patients positive for either test should be promptly treated.

## 1. Introduction

The risk of latent tuberculosis reactivation in rheumatoid arthritis (RA) patients is a perpetual concern among rheumatologists. Patients with RA have a 4-fold increased risk of tuberculosis (TB) infection compared with the general population [1]. Persons with latent TB infection (LTBI) are infected with* Mycobacterium tuberculosis* but are not clinically ill and have no symptoms or evidence of active TB. Thus all patients with suspected LTBI must be screened appropriately for active TB before treatment. Many RA patients have long-term treatment with corticosteroids and, more recently, with Biologic Response Modifiers (BRMs) that further increase their risk of LTBI reactivation. Several studies have associated corticosteroid use with heightened reactivation risk; also antitumor necrosis factor-*α* (TNF-*α*) therapy is strongly associated with reactivation of TB in patients with RA [2–9].

Most cases of TB in patients receiving immunosuppressive therapy have been attributed to reactivation rather than* de novo* infection with* Mycobacterium tuberculosis* [2, 10, 11]. An increase in the incidence of TB has been reported in industrialized cities populated by large number of immigrants from developing countries [12].

There is no diagnostic gold standard for LTBI. Currently, two vastly different methods of testing are available: the traditional Tuberculin Skin Test (TST) and the newly developed Interferon Gamma Release Assays (IGRAs). The TST measures type IV hypersensitivity in response to purified protein derivative (PPD), which contains a mixture of antigens of* Mycobacterium tuberculosis* that are also present in* Mycobacterium bovis*, and other nontuberculous mycobacteria. Using an immunologic approach, IGRAs measure antigen-specific interferon *γ* secretion by peripheral blood CD4+ lymphocytes in response to* in vitro* stimulation with ESAT-6, CFP-10, and TB7.7 peptides. IGRAs have been approved by the CDC as an alternative screening strategy to TST for the diagnosis of LTBI [13]. Currently the available IGRAs are the Quantiferon TB Gold (QFT-G) and the T-SPOT TB assay (ELISPOT-TB test).

Neither the TST nor the IGRAs have been proven to be 100% accurate. TST's main weakness appears to be its low sensitivity in immunosuppressed patients because of their deficient cell-mediated immunity and/or chronic use of immunosuppressants [14–20]. However, the clinical utility of IGRAs as a sole test for the detection of LTBI in immunocompromised patients is debatable [21]. Clinical guidelines for LTBI screening prior to BRM use are well established, though evidence based guidelines for rescreening during treatment after negative initial testing are less well defined.

The objective of this retrospective study is to compare an IGRA (Quantiferon TB Gold) test with the traditional TST in a high risk RA population and formulate a safe, practical, and cost-effective strategy for the diagnosis of LTBI in RA patients.

## 2. Material and Methods

### 2.1. Patients

**Figure 1:**
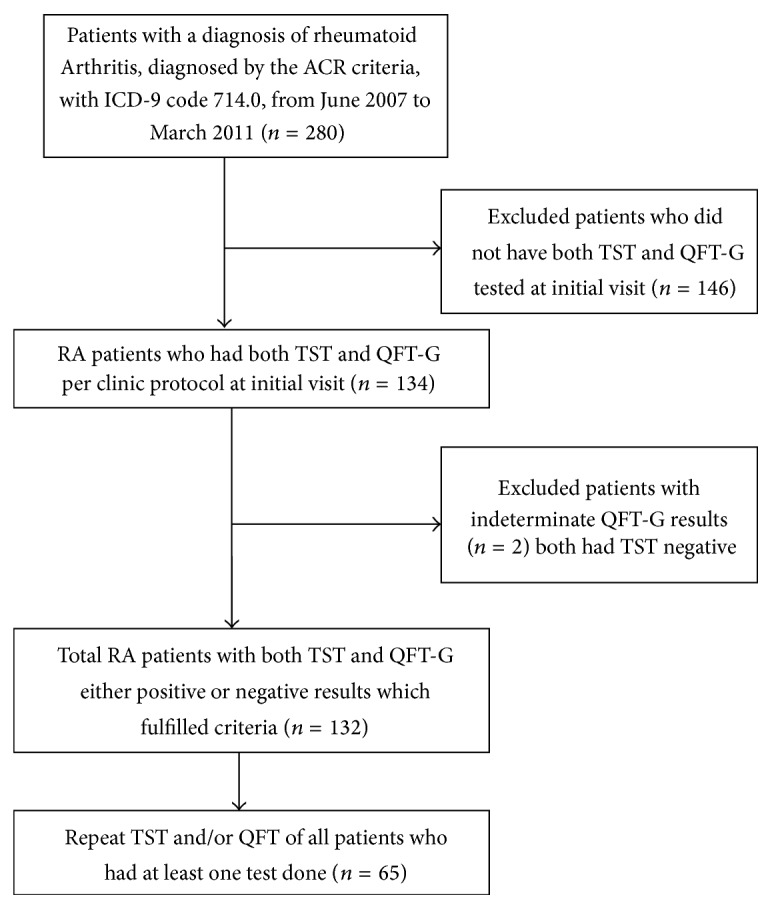
Method of selection of patients.

After the institutional review board (IRB) approval, 280 RA patients from an urban teaching hospital in New York were identified from June 2007 to March 2011 via Electronic Medical Record (EMR) chart review using ICD-9 code 714.0 (Figure 1). Patients had to fulfill the American College of Rheumatology (ACR) 2010 RA classification criteria [22]. 134 RA patients who had both TST and QFT-G tests for LTBI simultaneously performed were included in the study. Chest radiography was not performed for all patients as screening for LTBI irrespective of them going to be started on biologics or not. A retrospective chart review for each identified subject was conducted. The following data was collected: age, sex, comorbidities, ethnicity, disease duration, RA medications (DMARDs, steroids, and BRMs), and TST and QFT-G test results. Data regarding the BCG vaccination status was not available. All subsequent follow-up TST/QFT-G test results were retrieved.

### 2.2. TST Procedure

TST was performed by the Mantoux technique. 0.1 mL of 5 tuberculin units (TU) of PPD was injected intradermally into the dorsal or volar surface of the patient's forearm. Tests were read 48 to 72 hours after administration. Induration, not erythema, was measured and recorded; the transverse diameter of induration was recorded in millimeters. Tests were performed and read by 3 trained nurses in the outpatient department to maintain consistency and reduce bias. TST was considered positive if the induration was ≥5 mm as per the current CDC recommendations for RA [23].

### 2.3. QFT-G Assay

5 mL of heparinized whole blood was collected from patients by venipuncture. It was incubated for 16 to 24 hours at 37°C (99°F) in a humidified atmosphere. The test had a negative control (nil well, which has whole blood without antigens or mitogens); a positive control (mitogen well, which has whole blood stimulated with mitogen phytohemagglutinin); and 2 sample wells, which have whole blood with* M. tuberculosis*-specific antigens. IFN-*γ* levels in the nil well are subtracted from the results of the mitogen and the sample wells because they are considered background for the same. The results were considered positive if the IFN-*γ* concentration in the sample wells was 0.35 IU/mL or greater (after subtraction of the nil well value) and negative if the IFN-*γ* concentration in the sample wells was less than 0.35 IU/mL (after subtraction of the nil well value) and if the positive control was 0.5 IU/mL or greater. The results were considered indeterminate if both antigen-stimulated sample wells were negative and if the value of the positive control well was less than 0.5 IU/mL (after subtraction of the nil well value). A positive result suggested that* M. tuberculosis* infection was likely and a negative result suggested that infection was unlikely. An indeterminate result suggested that the QFT-G test results cannot be interpreted because of low mitogen response or high background response.

### 2.4. Treatment

All patients with either a positive TST or QFT-G as defined above were considered positive for LTBI. Only patients with positive screening for LTBI had chest radiography performed to rule out active TB or its sequelae. Once it was ruled out, patients were treated with isoniazid for 9 months.

### 2.5. Statistical Analysis

All patients with indeterminate QFT-G (*n* = 2) were excluded from the statistical analysis.

The kappa coefficient (*κ*) was calculated to determine the concordance between the two tests: TST and QFT-G. The strength of the agreement was considered “poor” for *κ* ≤ 0.20, “low-moderate” for 0.20 < *κ* ≤ 0.40, moderate for 0.40 < *κ* ≤ 0.60, “substantial” for 0.60 < *κ* ≤ 0.80, and “optimal” for 0.80 < *κ* ≤ 1. Baseline demographics and disease characteristics were summarized using descriptive statistics. We also analyzed the yield of both QFT-G and TST on initial and subsequent tests. All conversions, both TST and QFT-G, were carefully evaluated for clinical significance.

## 3. Results

Two hundred and eighty consecutive RA patients from an inner city teaching hospital outpatient rheumatology clinic were identified. These patients fulfilled the American College of Rheumatology (ACR) criteria and were coded for an ICD-9 code of 710.0. Of these 280 patients, 134 patients met the criteria of having both TST and QFT-G tests done. Two patients with indeterminate QFT-G were excluded from the analysis (Figure 1).

### 3.1. Patient Characteristics

A total of 132 patients were enrolled, including 115 (87.1%) females and 17 (12.8%) males with age ranging from 22 to 88 (average age 54.9 (standard deviation (sd) 12.2)), among which 100 (75.7%) were Hispanic and 32 (24.2%) were non-Hispanics reflecting the demographics of the population served by the hospital. 33 (25%) patients had a history of significant corticosteroid use, which was defined as 15 mg of prednisone for more than 1 month as per the CDC recommendations [24]. Additionally, 120 (90.9%) had been prescribed traditional DMARDs that included methotrexate, hydroxychloroquine, sulfasalazine, and leflunomide. 49 (37.1%) patients were prescribed BRMs subsequently. Biologics included predominantly TNF-*α* inhibitors (infliximab, etanercept, adalimumab, certolizumab pegol, and golimumab). The IL-1 receptor inhibitor anakinra, the IL-6 receptor inhibitor intravenous tocilizumab, the T cell costimulation inhibitor abatacept, and the B cell depleting agent rituximab were also prescribed for selected patients (Table 1).

### 3.2. Baseline Evaluation of TST and QFT-G

The positivity of QFT-G is 17.4% (23), whereas positivity of TST is 31.8% (42).

50 (37.8%) patients were diagnosed with LTBI with either a positive TST 42 (31.8%) or QFT-G 23 (17.4%). 15 (11.4%) were positive and 82 (62.1%) were negative for both tests. The agreement between TST and QFT-G was 73.5% (Kappa 0.305, CI = 95% 0.147–0.463, *p* = 0.081). A Kappa value of 0.305 represents a low-moderate agreement between the two tests. Sensitivity of QFT as compared to TST is 35.7%, whereas specificity is 91%. Moreover, sensitivity of TST as compared to QFT is 65%, whereas specificity is 75%. The yield on the initial TST screening was 31.8%, whereas QFT-G was 17.4% (Table 2).

### 3.3. Follow-Up Evaluation of TST and QFT-G

Of the 132 patients included, 65 had follow-up with at least one screening test for LTBI, either TST or QFT-G, with a mean follow-up of 2.41 yrs (SD = 1.24). Of the 82 patients who were negative for both tests, that is, “double negatives,” 55 patients had at least one follow-up screening for LTBI, either QFT-G or TST, and the mean follow-up was 2.32 yrs (SD = 1.33). In the 48 patients who had a second TST performed, the yield was 13%, whereas in the 27 patients who had TST performed for a third consecutive time the yield fell to only 4% (Table 3).

Seven patients converted from a negative TST to a positive TST on yearly follow-up testing, 2 of which were on biologics, although none of the patients had a history of significant corticosteroid use. All seven patients had QFT-G negative at the initial visit and no subsequent QFT-G performed during the follow-up visits. None of these patients developed clinical tuberculosis (reactivation or* de novo* infection) and the conversion may signify restoration of the host immune response, as previously reported in other diseases also associated with immune dysregulation [25]. None of the patients on follow-up had an indeterminate QFT-G.

31 patients had QFT-G done for a second time with a yield of 10% while in 6 patients with QFT-G done for a third time the yield was 0% (Table 3). Three patients converted from a negative QFT-G to a positive QFT-G. Of the three, 2 had an initial negative TST and the third had a positive TST tested at the same time at an outside facility.

Only 1 patient who converted was on biologics and had a history of significant corticosteroid use. Interestingly, one patient remained TST negative at the time of QFT-G conversion.

The 2 patients with indeterminate tests that were excluded from the analysis had a negative TST at the same time as the indeterminate results and follow-up QFT-G tests at 1 and 4 months, respectively, came back negative. None of the patients developed active TB.

## 4. Discussion

The objective of this study was to compare TST and IGRA (QFT-G) as screening tools for the detection of LTBI in RA patients. This comparison is clinically important since reactivation of LTBI in patients receiving BRMs, especially TNF inhibitors, has been shown to confer increased morbidity and mortality [26, 27]. Moreover, TB screening before starting BRMs reduces the incidence of reactivation of latent TB infection by up to 85% [26, 27]. A well-recognized problem for the diagnosis of LTBI is the absence of a diagnostic gold standard and thus sensitivity and specificity of TST and IGRA cannot be directly measured.

TST has several limitations. It requires two visits to a health care facility and is subject to human errors [28, 29]. Furthermore, it may be affected by a previous BCG vaccination and the immune status of the person tested [15–17, 30]. Its sensitivity may be low in patients with RA because of RA's immune-mediated pathogenesis [28, 31–33] or due to the immunomodulators used for its treatment. Its specificity may be compromised because of cross-reactivity with nontuberculous mycobacterial infection [29, 31, 32, 34, 35]. However, IGRAs have their own limitations. Technical errors may interfere with the quality of the assay and its interpretation. The test cannot differentiate between active and latent TB, which decreases its specificity. Furthermore, the impaired cellular response consequent to active TB diminishes the IGRAs sensitivity.

Several studies have compared TST with IGRAs. Some have concluded that IGRAs are more useful than TSTs because of greater sensitivity and avoiding the confounding factor of prior BCG vaccination [36–38]. Overall agreement between 2 tests in various studies was around 56% to 72% and Kappa values around 0.22 to 0.42 [4, 20, 39–42]. Some experts have recommended serial testing of RA patients [41, 42]. In some patients, especially from endemic regions, a chest film, in addition to TST/IGRA, is required [43]. French guidelines recommend the need to have systematic queries for latent TB or past untreated TB and BCG vaccination for diagnosing LTBI [44]. As per the British guidelines, tuberculin tests are not recommended and the risk of TB needs to be assessed through clinical history and examination, chest X-ray, and, when required, use of the algorithm assessing the risk of TB versus the risk of chemoprophylaxis by a TB specialist [45].

**Figure 2:**
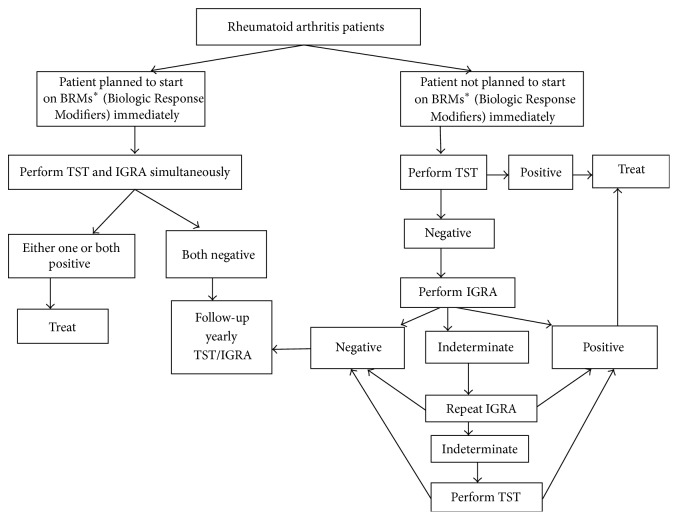
Proposed screening strategy for LTBI patient.

We propose what we think it may be a safe, rational, and cost-effective strategy for LTBI screening in RA (Figure 2). After a detailed history for active and latent TB along with physical exam, we recommend LTBI testing of all patients with RA, independent of planned use of BRMs. Studies have proven better outcomes in RA patients on anti-TNFs with TB screening [26, 46]. If the patient has high disease activity and/or evidence of radiographic progression at presentation and he/she needs to be promptly started on BRMs, baseline testing with TST and one IGRA should be performed concurrently. If either test is positive, the patient should be treated with antimicrobial agents according to the local protocol for LTBI treatment. Thereafter BRMs can be initiated when the treating clinician deems appropriate. If both tests return negative, BRMs can be initiated with repeat test performed yearly during follow-up appointments.

For less severe cases of RA, a two-step approach can be implemented that may have cost-saving advantages and may be easier to implement in health care systems with limited access to IGRAs. Providers can perform the TST as a first step since the yield of the initial TST in our study is higher and it is a universally available relatively inexpensive test. If positive, patient should be considered as having LTBI and be promptly treated. If, however, the initial TST is negative then an IGRA test should be performed. If the IGRA is positive, patient is considered again as having LTBI and, again, should be treated. If the IGRA is negative then the patient requires yearly follow-ups with TST/QFT-G. Monitoring LTBI serially in RA patients on BRMs is indicated [47].

In our study the yield of yearly follow-ups with TST was higher than QFT-G. There is data immunosuppressive therapy negatively affecting QFT-G function [20]. Considering the above and the costs of IGRAs versus TST, we recommend serial yearly screening with TST of double negative patients on initial screen.

Repeat testing should capture the immune-conversions that may occur after correction of the underlying immune dysfunction that occurs with DMARD and BRM treatment, reactivation, and, also,* de novo* exposures to* M. Tuberculosis*.

For patients with indeterminate IGRAs, we may consider repeating the IGRA within 3 months. If indeterminate for the second time, a TST should be performed and its result could be considered diagnostic. In patients with history suggestive of higher risk of TB or from an endemic area, CXR should also be used as a screening tool.

In another study, indeterminate results were less frequently reported with QFT-G than with T-SPOT TB; also no factor like steroid use or immunosuppressant use was associated with indeterminate results [48].

Similar studies have been performed in other high risk populations, such as in patients infected with HIV, where Ramos et al. have also suggested a two-step approach to LTBI testing similar to our proposed strategy [49]. A different approach was recommended in high TB burden areas with HIV patients or high risk immigrant population and where only TST-positive patients who should be screened again with IGRAs [50, 51]. In countries where the TB prevalence is intermediate and the BCG vaccination is mandatory at birth, a TST-only strategy could lead to unnecessary treatment with antibiotics. In this context, a strategy based on QFT-G results, regardless of TST results, seems effective to safely prevent TB in arthritis patients undergoing anti-TNF treatment [52]. In low TB incidence populations, researchers have also suggested this two-step approach, whereas only the TST positives are screened with IGRAs in order to decrease the number of false positives [53]. In high risk ESRD on HD patients, IGRAs are considered to be more accurate than TST according to a study by Lee et al. [54].

We believe our strategy to be safer, since missing cases of LTBI may lead to TB reactivations and, possibly, deaths that could be prevented with our comprehensive screening strategy. The additional cost and use of health care resources can be mitigated with our 2-step strategy whenever adequate time is available.

Our study has several limitations, mainly influenced by the fact that it is a retrospective study. The main limitation is the absence of individual BCG vaccination data. The hospital serves a dynamic, largely foreign-born population and BCG vaccination is a type A recommendation (where the country currently recommends BCG vaccination for everyone at a certain age (e.g., BCG at birth or for school-age children, etc.)) in the countries of origin [55]. Despite our efforts it was impossible to collect BCG vaccination data as the patients were unaware of their status and could not remember remote childhood events and there was no documentation available. Since these are all adult patients and BCG would have been given in an early age, theoretically the protective effect is known to wear out in 10 to 15 years. Also there have been studies in high risk populations that nontuberculous mycobacteria and prior BCG vaccination have minimal influence on TST results [56].

Additionally, our study was conducted in a high risk immigrant population and the results may not be easily generalized in low risk, low TB prevalence areas. However, we think the recent explosion of travel, immigration, and population shifts make our data and conclusions relevant. A recent prospective study proposed a similar strategy by comparing how likely positive TST and T-SPOT.TB results predict risk factors for tuberculosis in a predominantly immigrant patient population at risk of latent TB infection (LTBI) and with rheumatologic conditions requiring immunomodulatory therapy. They found that the combined use of TST and T-SPOT.TB appeared to be a reasonable diagnostic strategy to evaluate for LTBI in the rheumatology, as some TST(+)/TSPOT.TB(−) results were unlikely to be false-negatives [57]. Our strategy, if validated by other prospective studies, could simplify decision making by clinicians and would allow all RA patients, regardless of the presence of risk factors for LTBI, to safely benefit from the recent advances in RA management.

## Figures and Tables

**Table 1:** Baseline characteristics.

Characteristics	Positive TST and QFT-G	Positive TST and negative QFT-G	Negative TST and positive QFT-G	Negative TST and QFT-G	Total
Avg. age (years)	59.8	61.0	54.5	53.5	54.9
Std. deviation (years)	13.1	11.2	13.8	11.3	12.2
Male, *n* (%)	3 (2%)	1 (1%)	1 (1%)	12 (9%)	17 (13%)
Female, *n* (%)	12 (9%)	7 (5%)	26 (20%)	70 (53%)	115 (87%)
Hispanic, *n* (%)	13 (10%)	7 (5%)	22 (17%)	58 (44%)	100 (76%)
Non-Hispanic, *n* (%)	2 (2%)	1 (1%)	5 (4%)	24 (18%)	32 (24%)
SS^*∗*^, *n* (%)	6 (5%)	3 (2%)	9 (7%)	15 (11%)	33 (25%)
DMARDs^*∗*^, *n* (%)	13 (10%)	7 (5%)	25 (19%)	75 (57%)	120 (91%)
BRMs^*∗*^, *n* (%)	7 (5%)	2 (2%)	12 (9%)	28 (21%)	49 (37%)
No drugs, *n* (%)	1 (1%)	0 (0%)	1 (1%)	6 (5%)	8 (6%)
DMARDs^*∗*^ + SS^*∗*^ + BRMs^*∗*^, *n* (%)	5 (4%)	2 (2%)	8 (6%)	19 (14%)	34 (26%)
DMARDs^*∗*^ + BRMs^*∗*^, *n* (%)	7 (5%)	2 (2%)	12 (9%)	28 (21%)	49 (37%)

^*∗*^SS: history of significant steroid use, DMARDs: disease modifying antirheumatic drugs, BRMs: Biologic Response Modifiers, and *n*: number.

**Table 2:** Baseline evaluation of TST and QFT-G.

Screening test	TST positive number (%)	TST negative number (%)	Total number (%)
QFT-G positive	15 (11.4%)	8 (6%)	23 (17.4%)

QFT-G negative	27 (20.4%)	82 (62.1%)	109 (82.6%)

Total	42 (31.8%)	90 (68.2%)	132 (100%)

**Table 3:** Follow-up evaluation of TST and QFT-G with comparison of yields.

	Number of positive	Total number of patients tested	Yield (%)	Medications number (percent)
TST 1	42	132	31.8%	33 (25%) SS^*∗*^, 49 (37%) BRMs^*∗*^
TST 2	6	48	13%	11 (23%) SS^*∗*^, 16 (33%) BRMs^*∗*^
TST 3	1	27	4%	6 (22%) SS^*∗*^, 10 (37%) BRMs^*∗*^
QFT-G 1	23	132	17.4%	33 (25%) SS^*∗*^, 49 (37%) BRMs^*∗*^
QFT-G 2	3	31	10%	15 (48%) SS^*∗*^, 12 (38%) BRMs^*∗*^
QFT-G 3	0	6	0%	1 (17%) SS^*∗*^, 3 (50%) BRMs^*∗*^

^*∗*^SS: history of significant steroid use; BRMs: Biologic Response Modifiers.
